# Author Correction: DenRAM: neuromorphic dendritic architecture with RRAM for efficient temporal processing with delays

**DOI:** 10.1038/s41467-024-48315-z

**Published:** 2024-05-07

**Authors:** Simone D’Agostino, Filippo Moro, Tristan Torchet, Yiğit Demirağ, Laurent Grenouillet, Niccolò Castellani, Giacomo Indiveri, Elisa Vianello, Melika Payvand

**Affiliations:** 1 Institute of Neuroinformatics, University of Zurich and ETH Zurich, Zurich, Switzerland; 2 CEA-Leti, Université Grenoble Alpes, Grenoble, France

**Keywords:** Electrical and electronic engineering, Information technology

Correction to: *Nature Communications* 10.1038/s41467-024-47764-w, published online 24 April 2024

The original abstract of this Article was too long, which read

‘An increasing number of studies are highlighting the importance of spatial dendritic branching in pyramidal neurons in the neocortex for supporting non-linear computation through localized synaptic integration. In particular, dendritic branches play a key role in temporal signal processing and feature detection. This is accomplished thanks to coincidence detection (CD) mechanisms enabled by the presence of synaptic delays that align temporally disparate inputs for effective integration. Computational studies on spiking neural networks further highlight the significance of delays for achieving spatio-temporal pattern recognition with pure feed-forward neural networks, without the need of resorting to recurrent architectures. In this work, we present “DenRAM”, the first realization of a feed-forward spiking neural network with dendritic compartments, implemented using analog electronic circuits integrated into a 130 nm technology node and coupled with Resistive Random Access Memory (RRAM) technology. DenRAM’s dendritic circuits use RRAM devices to implement both delays and synaptic weights in the network. By configuring the RRAM devices to reproduce bio-realistic timescales, and by exploiting their heterogeneity we experimentally demonstrate DenRAM’s ability to replicate synaptic delay profiles, and to efficiently implement CD for spatio-temporal pattern recognition. To validate the architecture, we conduct comprehensive system-level simulations on two representative temporal benchmarks, demonstrating DenRAM’s resilience to analog hardware noise, and its superior accuracy compared to recurrent architectures with an equivalent number of parameters. DenRAM not only brings rich temporal processing capabilities to neuromorphic architectures, but also reduces the memory footprint of edge devices, warrants high accuracy on temporal benchmarks, and represents a significant step-forward in low-power real-time signal processing technologies.’

The abstract was truncated, which read

‘Neuroscience findings emphasize the role of dendritic branching in neocortical pyramidal neurons for non-linear computations and signal processing. Dendritic branches facilitate temporal feature detection via synaptic delays that enable coincidence detection (CD) mechanisms. Spiking neural networks highlight the significance of delays for spatio-temporal pattern recognition in feed-forward networks, eliminating the need for recurrent structures. Here, we introduce DenRAM, a novel analog electronic feed-forward spiking neural network with dendritic compartments. Utilizing 130 nm technology integrated with resistive RAM (RRAM), DenRAM incorporates both delays and synaptic weights. By configuring RRAMs to emulate bio-realistic delays and exploiting their heterogeneity, DenRAM mimics synaptic delays and efficiently performs CD for pattern recognition. Hardware-aware simulations on temporal benchmarks show DenRAM’s robustness against hardware noise, and its higher accuracy over recurrent networks. DenRAM advances temporal processing in neuromorphic computing, optimizes memory usage, and marks progress in low-power, real-time signal processing.’

The truncated abstract has been updated in both the PDF and HTML versions of the Article.

