# Supplementary material for: DenRAM: neuromorphic dendritic architecture with RRAM for efficient temporal processing with delays
Source: Nat Commun. 2024 Apr 24;15:3446. doi: 10.1038/s41467-024-47764-w (PMC11043378; doi:10.1038/s41467-024-47764-w)
Supplement: Supplementary file 1 — Supplementary information [file 41467_2024_47764_MOESM1_ESM.pdf]

Supplemental Materials:
DenRAM: Neuromorphic Dendritic Architecture with RRAM for Efficient Temporal Processing with Delays

Supplementary Note 1

We performed a thorough electrical characterization of a 16kbit RRAM array, with devices identical to the ones found in DenRAM. Devices are initially in the Pristine State, exhibiting large resistance, on the order of $G\Omega$. With a Forming operation - application of a positive voltage (>3V) to the Bit Line while the Source Line at ground - a conductive filament is formed in the device, programming the RRAM in the Low-Resistive State (LRS). Note that once the devices is Formed, it won't be possible for it to return in the Pristine State. We performed an adaptive Forming operation, meaning that we applied sequentially higher voltage pulses of the duration of 10ms until all the devices were formed. Most devices are Formed with a voltage pulse of about 4V. With a Reset operation - Bit Line at ground, Source Line with a positive voltage (2.3V) the conductive filament is broken, programming the RRAMs in the High-Resistive State (HRS). With a Set operation - Bit Line at a positive voltage [1.8-2.3]V, Source Line at ground - the devices can be programmed at the LRS. Varying the voltage applied on the Word Line [1.6-2.2]V during programming, different resistivity can be achieved, as shown in Figure S1. We also tested the Retention of our devices, by programming a 16 kb RRAM array at different resistive levels and periodically measuring the state of the array up to 24 h. Figure S2 reports the mean of the distributions over the 24 h retention test. We show that the highest conductance levels tend to slightly decrease in magnitude over time, while the lowest conductive levels are stable.

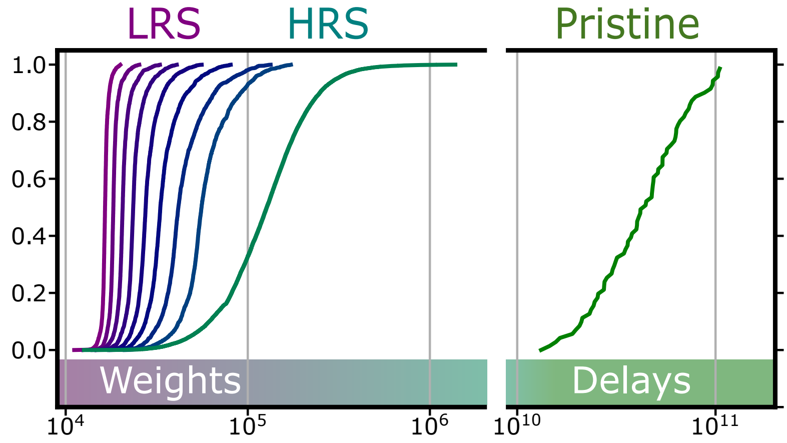

Figure S1: Characterization of the RRAM device and link to their role in DenRAM. Initially, RRAM devices are in the Pristine State. With a Forming operation, they are programmed in the LRS. With a Reset operation, they are programmed to the HRS. A Set operation programs the device back to the LRS. Delay RRAMs are left in the Pristine state, to maximize delays. Weight RRAMs are programmed in LRS or HRS.

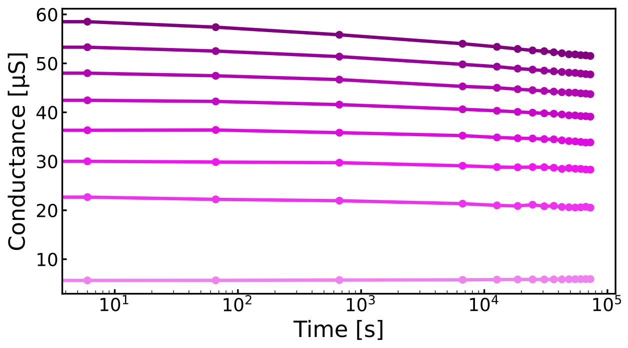

Figure S2: Retention measurements of the multi-level programming conditions. We plot the mean of the distributions obtained from a 16kb RRAM array, programmed at different average conductances. Measurement of the conductance level of the RRAM array is repeated over time, up to 24 h.

Supplementary Note 2

We introduced the dendritic circuit, the building block of the DenRAM architecture. An important component in this circuit is the Thresholding block, highlighted in Figure S3a, featuring a Schmitt Trigger and a Fall-Edge-Detector. The Schmitt Trigger circuit sets a threshold to the voltage at the Capacitor in the dendritic circuit, which relaxes from ground back to V_{ref}. The double threshold of the Schmitt Trigger allows for minimizing static power consumption. At the detection of the threshold, corresponding to the delay time produced by the Delay RRAM, the output of the Schmitt Trigger flips from high (1.2V) to low (0V). This triggers the Fall-Edge-Detector, built by a chain of Inverters and an OR circuit. The Fall-Edge-Detector produces the spike that is then fed to the output section of the dendritic circuit.
Another important element of DenRAM is the Leaky-Integrate-and-Fire neuron circuit (Fig. S3b). We design a simple LIF neuron taking an input current, and accumulating it on a membrane capacitor, thus increasing the membrane voltage V_{mem}. Transistor M1 is used to leak some of the current accumulated on the capacitor, and its bias Vlk can be tuned to control the leakage rate. An output section features a chain of inverters that detect the overcoming of the threshold by the membrane voltage V_{mem}. Such threshold crossing is favored by the positive feedback circuit involving transistor M3, which avoids the meta-stability of the membrane voltage. Transistor M2 is activated when the threshold voltage of the 2 inverters is crossed, grounding the voltage at the capacitor, and resetting the LIF neuron. Such a circuit is also assumed as the neuron model for the SRNNs system-level simulations.

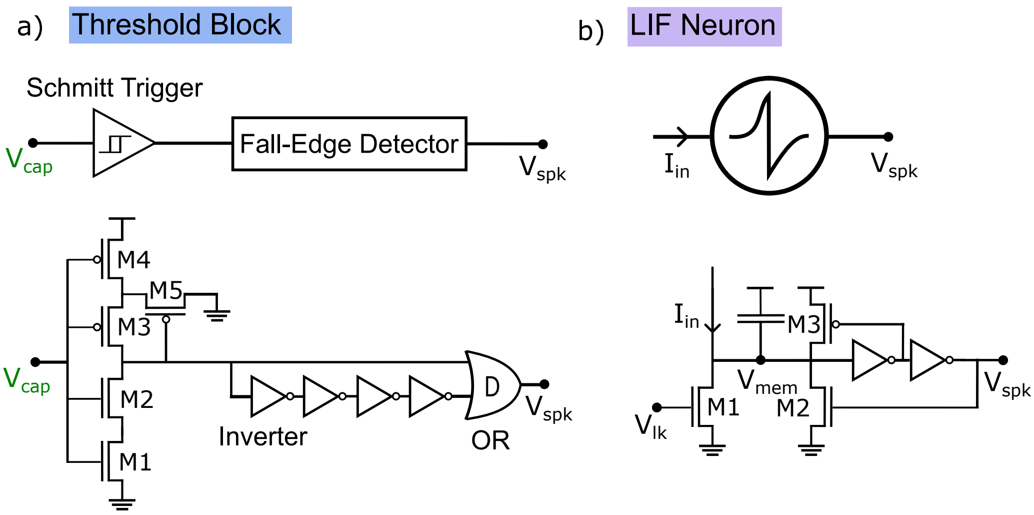

Figure S3: Circuits blocks of the DenRAM architecture. a) The Threshold Blok in the Dendritic Circuit features two sub-blocks: a Schmitt Trigger and a Fall-Edge-Detector. The schematics of the two sub-blocks are shown below. b) Leaky-Integrate-Fire neuron circuit. The circuit features a Capacitor, a biased transistor M1 controlling the leakage rate with the V_{lk} bias voltage, and an output section to produce the output spikes (V_{spk}). Transistor M2 resets the membrane voltage Vmem after a spike, and M3 provides positive feedback to avoid meta-stability when producing an output spike.

Supplementary Note 3

DenRAM architecture leverages spatio-temporal features of the inputs to perform computation, implementing coincidence detection (CD) as illustrated in Fig. 3c. To perform CD, temporally coincident spikes are assigned high weight, by setting the Weight RRAM to the LRS. However, it is also able to do the opposite: to set the weight of the dendritic circuit in HRS, so that coincidence is not detected.

Figure S4 shows how two spikes arriving very close in time can be separated by modulating the weight of the delays associated with the input of the two channels. Differently that in the experiment shown in Figure 3c, the weight associated with spike D_1 is set to HRS. In this way, the effect of the pair of coincident spikes D_1, D_2 on the output membrane voltage is lower and it prevents the neuron from spiking and thus detecting coincidence.

This mechanism can be critical if a group of spikes has to be correlated with another one without considering the influence of some previous spikes: in this case, CD and spike separation will be performed together by the network shifting one channel in time.

We also show an extension of the experiment shown in Figure 3d. In Figure S4, we report the Coincidence Detection window, i.e. the output activation of the post-synaptic neuron, as a function of the arrival time of input 2 (IN_2), for two different time constants. We show that the CD window increases as the time constant of the post-synaptic neuron increases: note that in the case of $\tau = 15ms$ the CD remains active in a window

of about 17ms, while the window shrinks at 10ms for $\tau = 10ms$. Notably, the CD window is centered at 58ms, corresponding to the delay D_1. This is thanks to the unique delay functionality of the dendritic branches of DenRAM. Such functionality is leveraged at the system level to perform heartbeat anomaly detection and keyword spotting with unprecedented accuracy.

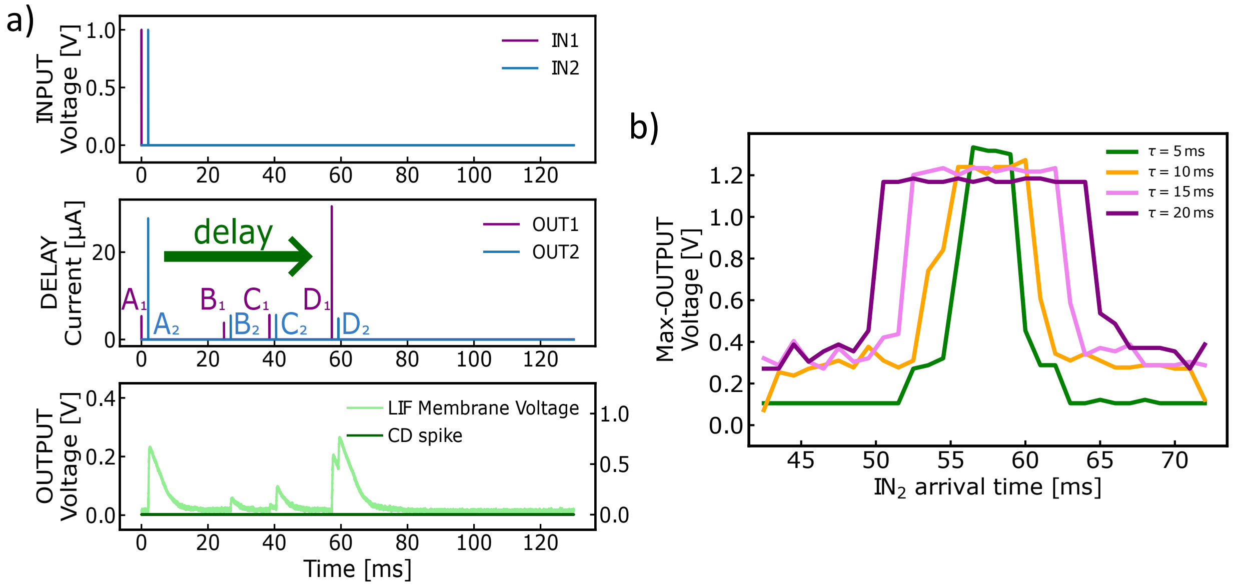

Figure S4: a) The dendritic architecture can also be used to separate spikes by the network that are not correlated but could lead to an output spike or change in the membrane voltage too significant; in this example, two spikes coming in with a delay of 1 μs have been separated thanks to delays, without they would have led to an output spike. b) Coincidence Detection window measured in DenRAM, as a function of the output neuron's time constant. With the same setup described in Figure 3, main text, we have repeated the Coincidence Detector experiment, changing the time constant of the output neuron.

Supplementary Note 4

We performed a thorough analysis of the ECG task, varying different parameters regarding the DenRAM architecture. This study, for such a simple architecture, involves the variation of two parameters: the standard deviation of the distribution used for sampling the delays and the number of delays per dendrites (i.e. the number of synapses). Through simulations, we found that for both the tasks analyzed in this paper, i.e. heartbeat arrhythmia detection (ECG) and spoken digit classification (SHD), the High Resistive Level was not sufficient to achieve high classification accuracy. HRS has a resistance of about $100k\Omega$, which coupled with the capacitor of $400fF$, implemented in DenRAM, produces delays that are not long enough for the analyzed tasks. This of course does not exclude the possibility that there might be other tasks that can be solved by shorter delays, for which the HRS resistance is a good match. It is interesting to analyze the effect of varying the distribution of the Delay RRAM (R_d) on the performance of DenRAM. Figure S5a shows the fit with a lognormal distribution of the Delay RRAM data ($\sigma = 0.5$), as well as how such distribution would look if the standard deviation would change. Based on these distributions, we performed system-level simulation on the ECG task fixing the number of synapses per dendritic branch in DenRAM. In Figure. S5b, we sweep the mean of the delay distribution with three standard deviation values, analyzing performance. Again, the green curve ($\sigma = 0.5$) is calibrated on hardware data, but the plot shows that a standard deviation of 0.75 is more beneficial when the mean of the delays is low. It is probably because of the long tail of the lognormal distribution with $\sigma = 0.75$, guaranteeing long delays despite a low average. Nonetheless, RRAM-calibrated DenRAM shows high accuracy (>95%) when the mean delay is higher than 20 ms.
Variability in the Weight RRAM [1] is also impacting the performance of DenRAM. As explained in the Method section, we apply noise-resilient training by applying a Gaussian noise during learning [2]. We investigate the performance of DenRAM and an SRNN performing at the same level (95%) with no variability in the weights. The Standard Deviation of the noise added to the weight is normalized by the largest weight in the layer where Gaussian noise is applied. Increasing the noise level in the DenRAM results in a modest decrease in performance, that only noticeable when the noise standard deviation is larger than 15%. In the SRNN instead, noise begins to decrease performance when the standard deviation is at 10%, meaning that DenRAM is more noise-resilient to the variability of weights. We also investigate the impact of the number of hidden neurons on the performance of the SRNN. Increasing the number of hidden neurons increases the memory footprint, but it also improves

classification accuracy and resilience to synaptic variability. In Figure S5d, we show the classification accuracy of the SRNN with either a 0% noise (ideal weights) or 10% noise applied to the weights. An SRNN with 32 hidden neurons is required to solve the task and match the performance of the DenRAM architecture. This network size is thus assumed for the Memory Footprint and Power Consumption comparisons in Figure 4b,c.

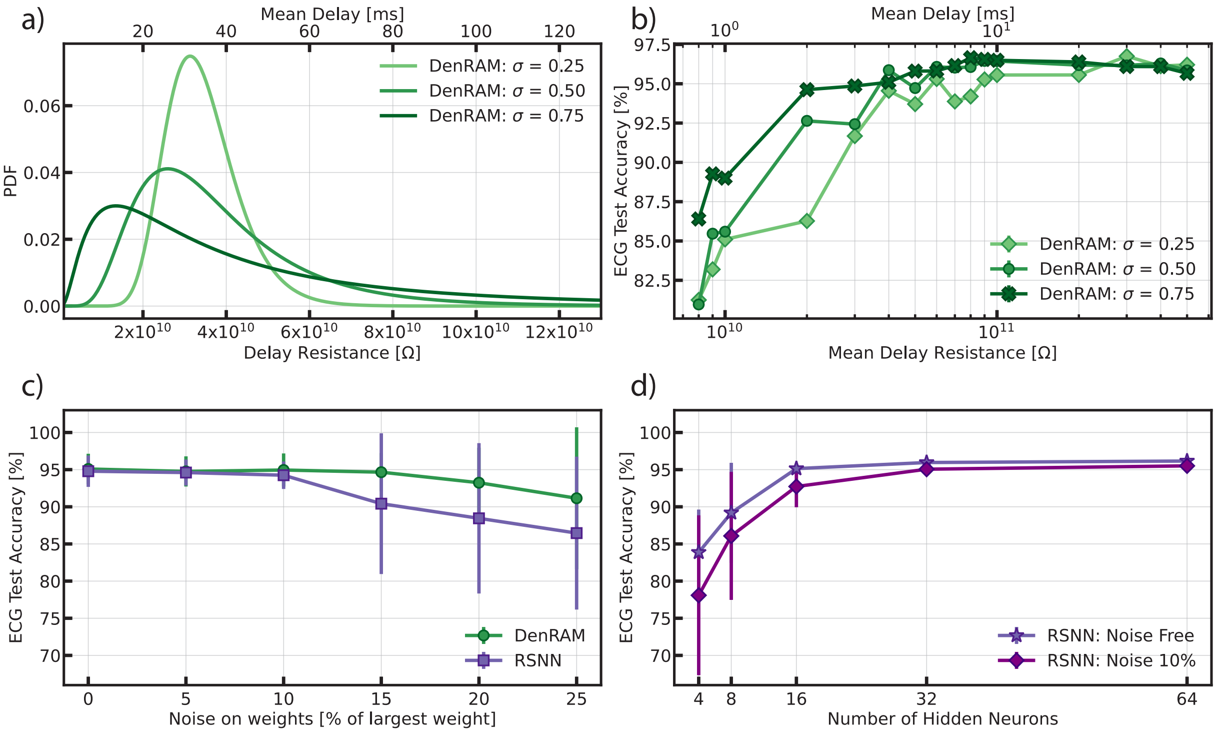

Figure S5: Ablation study on DenRAM applied on the ECG task. a) Lognormal distribution of the Delay RRAM (R_d). We denote σ as the standard deviation of the underlying normal distribution of RRAMs. The RRAM data are fit with $\sigma = 0.5$. b) Accuracy as a function of the mean of the Delay RRAM's distribution, for different distributions. Weights are subject to 10% noise. c) Accuracy of DenRAM and the SRNN as a function of the noise added on the weights, representing the variability of the Weight RRAMs. d) Impact of the number of hidden neurons on the performance of the SRNN, with and without RRAM-calibrated noise applied to the weights. In each plot, error bars represent the standard deviation over 10 trials.

We carry out a similar analysis of the SHD task using two networks with different number of inputs and different number of delays. (Fig. S6.). We first see that for low mean resistance, higher standard deviation helps the network to reach higher accuracy. For high mean resistance, the standard deviation importance is less clear. This behavior can be explained by the sampling of the log-normal, which is generated by sampling a normal distribution and then taking the exponential of the sampled value. Thus increasing the mean of the log-normal distribution while keeping the standard deviation of the underlying normal distribution will actually yield a distribution with increased standard deviation. Finally we see that the accuracy increases with the mean delay until 400 ms, past this value, the accuracy decreases, implying that there's an optimal mean delay in the range of 200 ms to 700ms.

Heterogeneity can not only manifest in DenRAM as the distribution of Delay-RRAM resistances but also in the different sizes in the dendritic branches. In a software implementation context, the "length" of the dendrites can be considered as the number of synapses per dendrite (which in our simulations is fixed). To implement length heterogeneity we can prune a given proportion of synapses according to the absolute value of the associated weights. This yields a network where the input channels are delayed a random number of times (instead of a fixed common number of times). Figure. S7a, shows the accuracy of DenRAM on the heartbeat anomaly detection task as a function of the proportion of removed synapses. In Fig. S7b, we show that we can prune up to 40% of the synapses with the lowest absolute weights and get tolerable accuracy drops of around 2% for both architecture sizes utilized in the keyword spotting task. Fig. S7c shows the resulting distribution of the number of synapses per dendritic branch with 10% (blue) and 20% (yellow) of the synapses pruned. The resulting distribution shows heterogeneity in the number of synapses per branch, similar to biology. With 40% of the synapses pruned, some branches have no synapses, which is equivalent to the case where inputs have different numbers of branches.

We further examine the effect of faulty RRAM devices on the classification accuracy of DenRAM. RRAM failure could occur by becoming stuck either in a High Resistive State (HRS) or a Low Resistive State (LRS). In

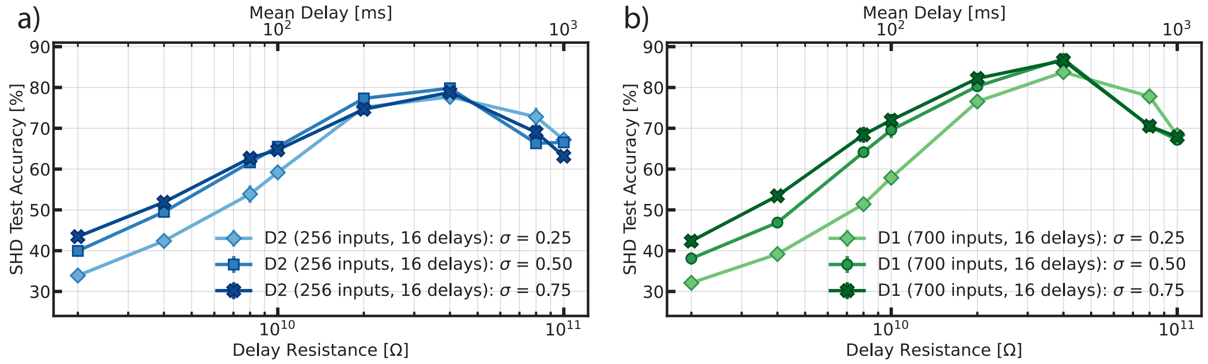

Figure S6: Ablation study on two DenRAM architectures applied on the SHD task. a) Accuracy as a function of the mean of the Delay RRAM's distribution, for different distributions, using 256 inputs and 8 delays. b) Same as a), using 700 inputs and 16 delays. In both plots, we denote σ as the standard deviation of the underlying normal distribution of RRAMs. Weights are subject to 10% noise. Error bars represent the standard deviation over 3 trials.

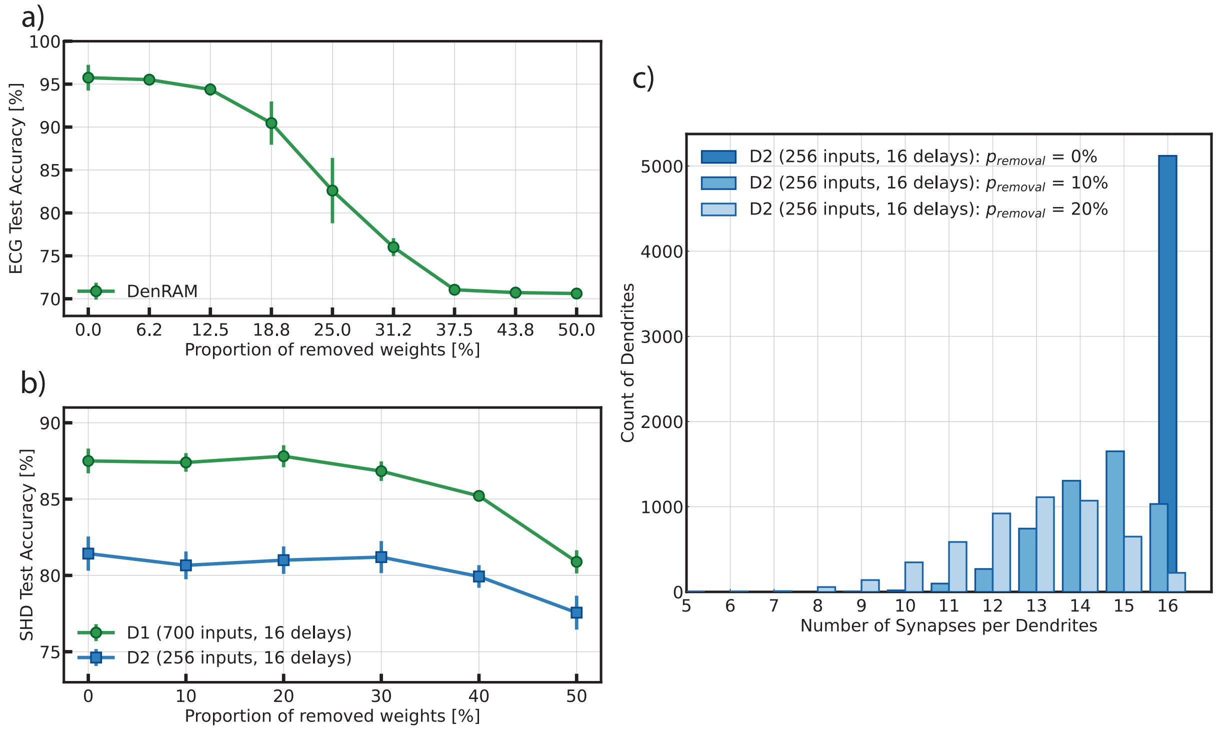

Figure S7: Effect of heterogeneity in dendritic branch size. a) Removing the synapses with the smaller associated absolute weight for the ECG task. We sweep the percentage of synapses removed. Error bars represent the standard deviation over 10 trials. b) Same as a) but on SHD task. Error bars represent the standard deviation over 3 trials. c) Number of synapses per branch after removal for architecture D2, for different removal proportions, denoted as $p_{removal}$.

the following analysis, we assume the two failure types to have equal probability. We perform RRAM simulation on the networks trained for Fig. 4 of the main text, artificially introducing failures in the Weight RRAMs. First, we gauge the effect of faulty RRAMs on the classification accuracy, assuming that the model is trained without any prior knowledge of the RRAM failure. The results are reported in Fig. S8 under PTS (Post Training Stuck). We find that the larger network trained on the SHD task (Fig. S8b) is more resilient to PTS than the smaller network that was used for the ECG task (Fig. S8a). We then analyze the performance of DenRAM when the network is re-trained aware of the faulty devices. The results are reported in Fig. S8 under SAT (Stuck Aware Training). We find that the accuracy on the ECG task is almost fully restored to the original case without RRAM failures while accounting for up to 25% faulty devices. Similarly, DenRAM applied to the SHD task shows little accuracy degradation for up to 30% of faulty RRAM devices. These results confirm that DenRAM is not only energy-efficient and parameter-efficient but also resilient to faulty RRAM devices, especially if the network is aware of the device yield.

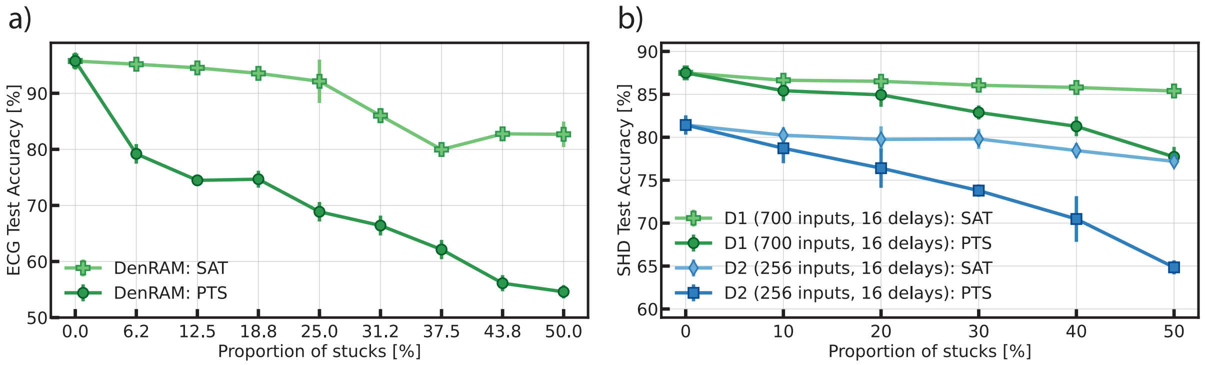

Figure S8: Effect of faulty weights in the ECG task and SHD tasks. PTS: post-training stuck, stuck devices are acknowledged only during inference; SAT: stuck-aware training, stuck devices are acknowledged during training. a) Analysis of network resilience on the ECG task. Error bars represent the standard deviation over 10 trials. b) Same as a) but on the SHD task and for two different network sizes (D1 and D2). Error bars represent the standard deviation over 3 trials.

Supplementary Note 5

As explained in the main text, the DenRAM architecture delays each input channel with a population of n_{delays}, randomly sampled from a fixed log-normal distribution which models the measurements shown in Fig. 2d. Here, we are applying the inputs from the Spiking Heidelberg Digit (SHD) dataset to the DenRAM architecture. Fig. S9a shows sub-sampled version of the digits “8”, “18”, and “17” of the SHD dataset, from 700 input channels to 256 chanels. Each of the channels in these examples are delayed $n_{delays} = 16$ times in the case shown in the figure. Consequently, the output neurons receive $n_{in} \times n_{delays}$ spike trains (in this case $256 \times 16 = 4096$ delayed inputs, shown in Fig. S9b. After we train the network, it learns to weigh each of these 4096 channels in order to classify the presented input. To analyze the weight-delay pair, we do a weighted average of the n_{delays} delayed channels for each input channel, giving rise to an aggregate representation in Fig. S9c. We observe that each input channel acquires a time-varying weight (illustrated in Fig. S9c) to solve the task. For instance in digit “8”, channels 150 to 200 exhibit a dynamic pattern, initially showing inhibition, then strong excitation, and finally returning to inhibition. Although digits “8” and “18” display similar patterns when delayed (see Fig. S9c), the network distinguishes them by utilizing different input channels, a phenomenon we term ’spatial segregation’. In the case of digit “17”, the selected input channels resemble those for digit “8”, yet the delays cover a more extended time range, indicating ’temporal segregation’. This suggests that while certain input channels are shared across different digits, the network differentiates them through the temporal distribution of these channels’ activations.

This dual strategy of spatial and temporal segregation enables the network to effectively distinguish between various inputs, even when they exhibit similar delayed signatures. In the illustration provided in Fig. S9d, the dynamic behavior of the output membrane voltage for the selected digits substantiates our observations. In the case of spatial segregation, it is evident that while the membrane voltage peaks for digits “8” and “18” occur simultaneously, their magnitudes are influenced by the specific inputs, which helps with their segregation. For example, in the case of digit “18” as the input, activation of channels 75 to 125 plays a key role to reduce the potentiation of neuron “8”, whose corresponding weights are negative, while increasing the potentiation of neuron “18”, whose correspoinding weights are positive. Therefore, the network correctly gives the maximum membrane potential to output neuron “18”, classifying digit ‘18’.

In the case of temporal segregation, when digit “17” is fed as the input, the variation in weight dynamics becomes a critical distinguishing factor. In this scenario, the response pattern for neuron “8” transitions from inhibition to excitation and back to inhibition, whereas for neuron “17”, the pattern is consistently excitatory, leading to a higher peak in its membrane potential, correctly classifying the input.

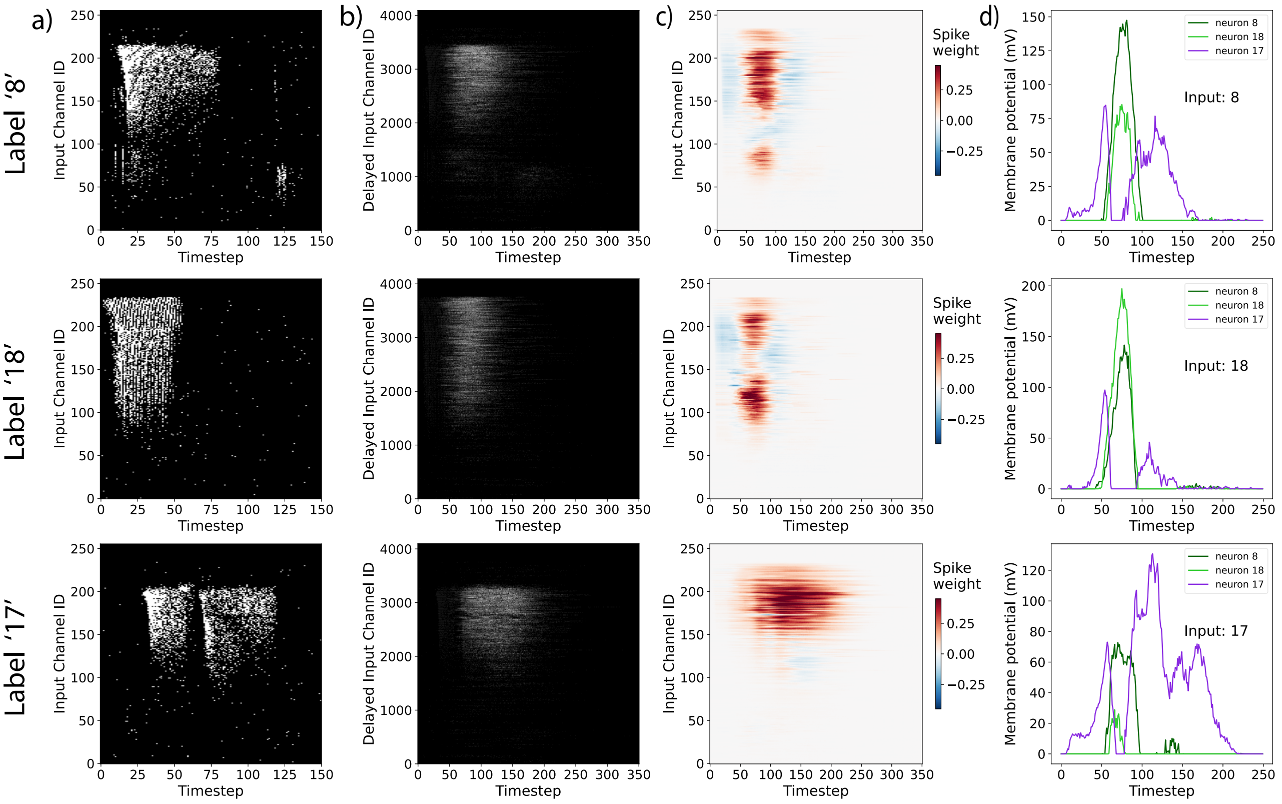

Figure S9: Analysis of the weights and delays for the D2 network (DenRAM with 16 delays per channel, using 256 input channels) . We focus on three classes from the SHD dataset: spoken digit “8”, “18” and “17”. a) Raster plots representing individual training samples for three classes. These original samples comprise 256 channels sampled from the 700 input channels from the original dataset, and span 150 timesteps. b) Raster plots illustrating time-delayed versions of the same samples after feeding into DenRAM. The DenRAM architecture generates time-shifted replicas of the original inputs, expanding them to 4096 channels (256 channels $\times$ 16 delays per channel) and 350 timesteps. c) After learning, for each channel, the 16 delayed version are weighted and summed, giving rise to an aggregated representation of weighted-delayed input for each channel, condensing back the 4096 channels to 256. Red-shifted color represent positive weights, and blue-shifted colors represent negative ones. The network learns to weight each aggregate-channel differently at different time steps. These plots present the weighting of the average spike train across all samples from the respective classes. d) Evolution of the output membrane potentials of the three classes when exposed to the respective inputs.

References

[1] Eduardo Esmanhotto, Tifenn Hirtzlin, Djohan Bonnet, Niccolo Castellani, Jean-Michel Portal, Damien Querlioz, and Elisa Vianello. Experimental demonstration of multilevel resistive random access memory programming for up to two months stable neural networks inference accuracy. *Advanced Intelligent Systems*, 4(11):2200145, 2022.

[2] Filippo Moro. Memristor-aware-training for resilient neural networks, 2023.
